# Resistome Analysis of *Campylobacter jejuni* Strains Isolated from Human Stool and Primary Sterile Samples in Croatia

**DOI:** 10.3390/microorganisms10071410

**Published:** 2022-07-13

**Authors:** Silvija Šoprek, Sanja Duvnjak, Gordan Kompes, Luka Jurinović, Arjana Tambić Andrašević

**Affiliations:** 1 Department of Clinical Microbiology, University Hospital for Infectious Diseases “Dr. Fran Mihaljević”, 10000 Zagreb, Croatia; ssoprek@bfm.hr (S.Š.); arjana.tambic@bfm.hr (A.T.A.); 2 Laboratory for Bacterial Zoonoses and Molecular Diagnostics of Bacterial Diseases, Department for Bacteriology and Parasitology, Croatian Veterinary Institute, 10000 Zagreb, Croatia; marjanovic@veinst.hr; 3 Laboratory for General Bacteriology and Mycology, Department for Bacteriology and Parasitology, Croatian Veterinary Institute, 10000 Zagreb, Croatia; kompes@veinst.hr; 4 Laboratory for Bacteriology, Poultry Centre, Croatian Veterinary Institute, 10000 Zagreb, Croatia; 5 School of Dental Medicine, University of Zagreb, 10000 Zagreb, Croatia

**Keywords:** *Campylobacter jejuni*, antimicrobial resistance, antimicrobial susceptibility testing, bioinformatics, whole genome sequencing, *Campylobacter jejuni* in primary sterile samples

## Abstract

Campylobacteriosis represents a global health challenge due to continuously increasing trends of antimicrobial resistance in *Campylobacter jejuni*. *C. jejuni* can sometimes cause life-threatening and severe systematic infections (bacteremia, meningitis, and other extraintestinal infections) with very few antibiotics left as treatment options. Bearing in mind that *C. jejuni* is the predominant species in humans, in this paper, we present a study of the *C. jejuni* differences in antimicrobial resistance and genotype distribution between strains isolated from stool and primary sterile sites. We compared the genomic data obtained through whole genome sequencing (WGS) and phenotypic susceptibility data of *C. jejuni* strains. Once antimicrobial susceptibility testing of *C. jejuni* strains was carried out by the broth microdilution method for six of interest, results were compared to the identified genotypic determinants derived from WGS. The high rate of resistance to fluoroquinolones presented in this study is in accordance with national surveillance data. The proportion of strains with acquired resistance was 71% for ciprofloxacin and 20% for tetracycline. When invasive isolates were analysed separately, 40% exhibited MIC values of ciprofloxacin higher than the ECOFFs, suggesting a lower flouroquinolone resistance rate in invasive isolates. All isolates demonstrated wilde-type phenotype for chloramphenicol, erythromycin, gentamicin, and ertapenem. A special focus and review in this study was performed on a group of *C.jejuni* strains found in primary sterile samples. Apart from demonstrating a lower resistance rate, these isolates seem genetically more uniform, showing epidemiologically more homogenous patterns, which cluster to several clonal complexes, with CC49 being the most represented clonal complex.

## 1. Introduction

The most recent reports confirm that campylobacteriosis remains the first most reported zoonosis in humans, and the most frequently reported foodborne illness in the European Union (EU) [1,2]. It represents a global health challenge [3] due to continuously increasing trends of antimicrobial resistance both in medicine and agriculture, as well as the financial burden of approximately 2.4 billion euros annually estimated for the EU by the European Food Safety Authority (EFSA) [2]. Bearing in mind that *Campylobacter jejuni* is the predominant species of human campylobacteriosis worldwide [4], it places this species as the focus of our study.

*Campylobacter jejuni* infection is usually characterised as a mild, self-limited disease from which patients usually recover fully within a week. The disease is commonly characterised by several symptoms, including watery or bloody diarrhoea, stomach cramps, nausea, headache, and high fever [4]. Regardless, it can sometimes cause life-threatening and severe systematic infections (bacteremia, meningitis, and other extraintestinal infections) with very few antibiotics left as treatment options [5,6,7,8,9,10,11].

For many years, various antibiotic resistance mechanisms and virulence factors were observed and studied, and yet not all were explained. One of the main factors influencing the increasing rates of antimicrobial resistance, especially to fluoroquinolones and macrolides, is the use of these antimicrobial agents in animal production [12]. Resistance to the fluoroquinolones is mainly due to target gene mutation in the quinolone resistance-determining region (QRDR) of the DNA gyrase and topoisomerase IV, but it can be conducted by the efflux mechanism too [13,14].

Macrolide resistance in Campylobacter is the result of the modification of the ribosome target binding site by mutation of the 23S rRNA, or changes in resulting proteins at the site rather than target methylation or enzymatic drug modification [15,16,17].

Mechanisms of Campylobacter resistance to some beta-lactams, such as ampicillin and some of the expanded-spectrum cephalosporins, are variable and not very clearly defined. Generally, the majority of Campylobacter strains are considered to be resistant to beta-lactam antimicrobial agents, especially the penicillins and narrow-spectrum cephalosporins, except for some carbapenems. The first gene encoding β-lactamase located on a chromosome in Campylobacter was described by Taylor et al., a class D β-lactamase [18,19]. The corresponding gene, from a human clinical isolate, was cloned and characterised coding a class D β-lactamase, OXA-61, conferring resistance to ampicillin, penicillin, and carbenicillin in *C. jejuni* [20,21]. Meanwhile, there were several enzymes described based on their differing activity against eight beta-lactams. Another mechanism for beta-lactam resistance is active efflux by efflux pumps. Several studies demonstrated a significant decrease in susceptibility in CmeABC-overexpressing mutants [22,23].

Multiple aminoglycosides-modifying enzymes, including aminoglycoside phosphotransferase types I, III, IV, and VII, aminoglycoside adenyltransferase, and 6-aminoglycoside adenyltransferase, are described in Campylobacter [24]. Aminoglycoside resistance is mediated by enzymatic modification that decreases the affinity of aminoglycosides for the rRNA A-site [25,26].

Resistance to tetracyclines in Campylobacter is conferred by the *tet*(O) gene, which is widely present in both *C. jejuni* and *C. coli* [27,28,29].

Chloramphenicol resistance is conferred by a plasmid-carried cat gene that encodes acetyltransferase, which modifies chloramphenicol in a way that prevents it from binding to ribosomes [30].

It is expected that whole genome sequencing (WGS) can increase the understanding of the underlying mechanisms of resistance together with all the variability connected to this species at the same time. It could be a valuable tool in routine laboratory work as a predicting method of resistance, especially when standard procedures fail. It could have a crucial impact in situations where the pathogen cannot be tested for susceptibility to antibiotics with standard procedures. New diagnostic approaches in the analysis of this demanding microorganism with the potential for the development of antimicrobial resistance make this species of special interest for this kind of study.

In this paper, we analyse resistance determinants in *C. jejuni* clinical isolates, specifically in invasive and stool isolates. Isolates were collected from three microbiological laboratories in Croatia. This study aims to compare the genomic data obtained through WGS sequencing and phenotypic data of *C. jejuni* strains, as well as to review differences in antimicrobial resistance from the perspective of different sources and thus invasiveness of isolated strains.

## 2. Materials and Methods

### 2.1. Preparing the Bacterial Isolates

#### 2.1.1. Campylobacter Isolates

For this study, *C. jejuni* strains from 45 patients having symptoms of diarrhoea were collected from 4 independent microbiology laboratories in Croatia.

A total of 45 strains, consisting of 10 strains found in primary sterile samples (9 from blood cultures and 1 from cerebrospinal fluid, samples ZGI01-ZGI10) and 35 found in stool were studied and analysed. All strains originating from primary sterile samples together with 10 strains from stool samples were collected in Zagreb (the University Hospital for Infectious Diseases “Dr. Fran Mihaljević”, samples ZG01-ZG10), another 10 strains from stool samples were received from Split (Public health Institute of Split and Dalmatia, samples ST2-ST12), 1 from Osijek (University Hospital Center Osijek, sample OS01), and 11 from Pula (Teaching Institute for Public Health County of Istria, samples PU01-PU11). Once received in the Laboratories of the Croatian Veterinary Institute, strains were streaked and sub-cultured on blood agar supplemented with 10% of defibrinated sheep blood (Columbia agar, Biomerieux, Marcy-l’Étoile, France) and incubated in microaerobic conditions (CampyGen, Thermo Scientific, Waltham, MA, USA) at 42 °C for 48 h.

#### 2.1.2. DNA Extraction

When reviving bacteria, strains were cultivated on blood agar supplemented with 10% of defibrinated sheep blood and incubated in microaerobic conditions overnight. DNA extraction was performed by taking a full loop of fresh culture and resolving it in 100 μL of PCR clean water. The culture was then treated using a NucleoSpin Microbial DNA Mini Kit (Macherey-Nagel, Düren, Germany) according to the manufacturer’s instructions.

#### 2.1.3. Species Confirmation/Identification

All strains were further subjected to species determination performed by a multiplex PCR assay [31]. This method detects genes from the five major clinically relevant *Campylobacter* species simultaneously.

#### 2.1.4. MLST

Sequence types (ST) and clonal complexes (CC) were determined using the WGS plugin in BioNumerics 8.1 version (BioMerieux, Applied Maths, Sint-Martens-Latem, Belgium). The minimum spanning tree and dendrogram were then constructed using advanced and UPGMA cluster analyses. MLST profiles are given in Table 1.

#### 2.1.5. Whole Genome Sequencing

The extracted DNA was prepared according to the MicrobesNG (Birmingham, UK) instructions and sent there for sequencing. Only 42 samples were of high enough quality for whole genome sequencing. The sequencing was run on an Illumina platform with a 250 bp paired-end output. The results were obtained as raw trimmed reads and assembled fasta files.

### 2.2. Genomics

#### 2.2.1. Whole Genome Sequencing

The basic bioinformatic analysis was provided by MicrobesNG (Birmingham, UK). All obtained reads were put through a standard analysis pipeline. The closest available reference genome was identified using Kraken, and the reads were mapped to this using BWA-MEM (Burrows–Wheeler Aligner) to assess the quality of the data. A de novo assembly of the reads was performed using SPAdes, and the reads mapped back to the resultant contigs, again using BWA mem to get more quality metrics. Additionally, an automated annotation was performed using Prokka v1.12 [32].

All but 7 samples were de novo assembled into fewer than 40 contigs (average for 35 samples was 20.7) with a genome size of approximately 1.7 kb and GC content around 30%. Samples ST9, ZG03, ZG08, ZG12, ZGI05, ZGI06, and ZGI10 were inter-species contaminated, but we used reference mapping (reference sequence NC_002163) to isolate only *C. jejuni* sequences.

#### 2.2.2. Antimicrobial Susceptibility Testing

Antimicrobial susceptibility testing (AST) was performed on strains revived and sub-cultured on blood agar supplemented with 5% of defibrinated sheep blood. It was carried out by the broth microdilution method for six antimicrobials of interest, following the European Committee on Antimicrobial Susceptibility Testing (EUCAST) guidelines, on EUCAMP3 microplates (Sensititer, Trek Diagnostic Systems Ltd. East Grinstead, West Sussex, UK). Susceptibility to erythromycin (ERY; 1–512 mg/L), ciprofloxacin (CIP; 0.12–32 mg/L), and tetracycline (TET; 0.5–64 mg/L) was determined using EUCAST epidemiological cut-off values (ECOFFs), while for ertapenem (ERTA 0.12–4 mg/L), gentamicin (GEN; 0.25–16 mg/L), and chloramphenicol (2–64 mg/L), EFSA cut-off values were used, as there is no available data by the EUCAST [33,34]. Epidemiological cut-off values were used for interpretative thresholds for resistance, identifying the non-wild-type strains defined as potentially harboring resistance mechanisms.

To ensure that the results were within the acceptable limits of quality control for susceptibility testing, the *C. jejuni* ATCC 33560 reference strain was used.

The phenotypic AST results in six antimicrobial agents, determined by broth microdilution, are presented in Table 2.

The WGS-derived antimicrobial resistance (AMR) was analysed on de novo assemblies using the publicly available service ResFinder 4.1 [35] provided and curated by the Center for Genomic Epidemiology. We analysed 42 assembled *Campylobacter* spp. genomes for chromosomal point mutations (also for all unknown mutations) with 98% threshold for %ID and 100% minimum length, and acquired antimicrobial resistance genes using the same restrictions. Genes are divided into two groups: the ones that were a 100% perfect match, and those with an identified mutation (100% length identity but 98–99.9%).

#### 2.2.3. Genotypic–Phenotypic Comparisons

The WGS-derived AMR was compared to the results of in vitro AST for six clinically relevant antimicrobial agents (erythromycin, ciprofloxacin, tetracycline, gentamicin, chloramphenicol, and ertapenem).

Concordance between methods was determined by comparing the genotypic detection of known resistance determinants against the phenotypic susceptibility results of each strain at a concentration equal to the ECOFF described by EUCAST.10 or EFSA.

Major errors were classified as those instances in which a strain was predicted to be resistant due to the detection of an AMR determinant in the genome but was phenotypically susceptible. Very major errors were classified as those instances in which a strain was predicted to be susceptible by the absence of an AMR determinant in the genome but was phenotypically resistant.

## 3. Results

### 3.1. PCR Identification and Epidemiological Relatedness

All of the 45 tested strains were identified as *C. jejuni* using PCR according to Wang et al. [31]. We also used the WGS data to determine multilocus sequence typing (MLST) sequence types (ST) for 42 strains that were sequenced. Twenty-two different STs were identified, as well as one ST not yet present in the PUBMLST database. Additionally, the ZGI10 allelic profile could not be determined on all loci (Table 1).

**Figure 1:**
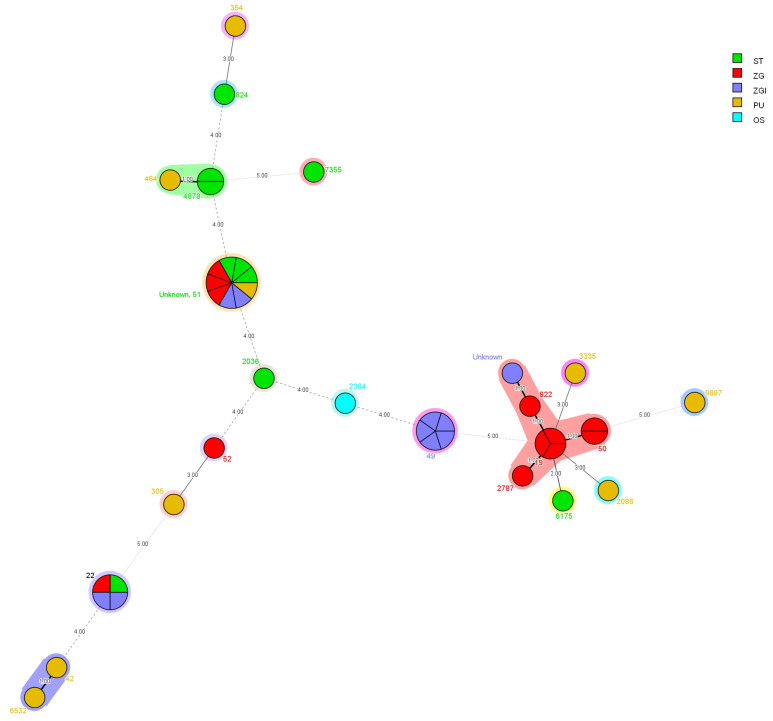
Minimum spanning tree (MST) showing relationships between tested strains according to MLST data (numbers by the samples represent the identified STs, whereas numbers on the lines connecting the samples represent the number of allelic differences between neighboring samples, and different colours represent the origin of the samples).

**Figure 2:**
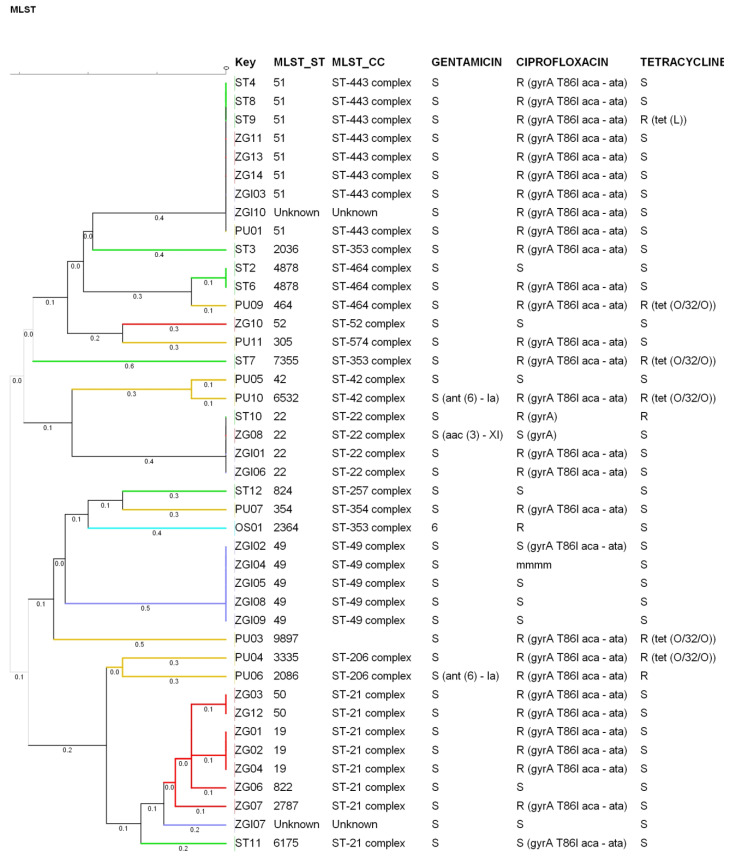
Dendrogram showing relationships between tested strains according to the MLST data and possible connection to identified antimicrobial resistance.

Minimum spanning tree and dendrogram data representations show that there are no epidemiologically relevant differences between strains isolated from different parts of Croatia (Figure 1 and Figure 2).

Among all the 45 strains tested, the proportion of strains with acquired resistance was 20% for tetracycline, and 71% for ciprofloxacin. Results from chloramphenicol, erythromycin, gentamicin, and ertapenem tests show that all tested strains were classified as wild-type strains (Table 2).

When referring to invasive isolates (isolate numbers ZGI01-ZGI10), in vitro susceptibility testing shows that 40% (4 of 10 isolates) of invasive isolates exhibit MIC values of ciprofloxacin higher than the ECOFFs, suggesting a lower fluoroquinolone resistance rate in invasive isolates. These isolates cluster to CC21, 22, 49, and 443 (and two unknown) clonal complexes. Clonal complex CC49 is the most represented, at 50%. Considering that the genotypic resistance rate to ciprofloxacin is very high in all tested samples (73.8%), these invasive strains show lower quinolone resistance. Genotypic resistance to ciprofloxacin is, as in almost all tested samples, linked to *gyr*A T86I mutation.

The correlation between the results of AST to six antimicrobial agents and WGS-derived antimicrobial resistance are presented in Table 2.

The phenotypic results of in vitro AST to six antimicrobial agents, determined by broth microdilution, are presented in (Table S1).

### 3.2. Genotypic Determination of AMR

Table 2 is showing determinants that are confirmed to a specific AMR. Four out of six antimicrobial agents resulted in both genotypic and phenotypic susceptibility (100%). Genotypically tested strains showed AMR in only three tested antimicrobial agents: streptomycin and an unknown aminoglycoside, ciprofloxacin (fluoroquinolone), and tetracycline (tetracycline). Resistance to ciprofloxacin (fluoroquinolone) was genotypically the most represented (77.5%). Resistance to tetracycline was detected in 6 out of 42 samples (14%), and resistance to streptomycin (aminoglycoside) in 3 out of 42 samples (7%) (Table 2). Genes connected to a possible beta-lactam resistance (*bla*OXA genes) were often identified in the tested samples, however, none of those are, as far as we know, put in direct connection to a specific antimicrobial beta-lactam agent. We identified four different *bla*OXA genes. The *bla*OXa-61 gene was identified in 18 samples (43%), *bla*OXA-184 in 11 samples (26%), and *bla*OXA-460 in one sample (2%), whereas *bla*OXA-461 was identified in 6 samples (14%) (Table S2).

### 3.3. Comparison between Phenotypic and Genotypic AMR

The phenotypic and genotypic antimicrobial predictions were highly correlated. In total, 6 strains out of the 42 tested on six different antimicrobial agents (2.4%) showed eight discordances between phenotypic and genotypic results (Table 3). Three strains showed genotypic resistance to ciprofloxacin, whereas they were phenotypically classified as strains without acquired resistance. Additionally, one strain was genotypically susceptible to ciprofloxacin and two to tetracycline but tested phenotypically as a strain with acquired resistance to the mentioned antibiotics.

#### 3.3.1. Resistance to Macrolides

Mutation in the 23S rRNA gene conferring reducing the susceptibility to erythromycin was not detected. All 39 of these isolates were also phenotypically determined as strains without acquired resistance to erythromycin (ECOFF > 8 mg/L) (Table 2). No discrepancies were found between the phenotypic and the genotypic profiles for erythromycin resistance in any of the 42 isolates tested with both methods.

#### 3.3.2. Resistance to Fluoroquinolones

Mutations in *gyr*A resulting in reduced susceptibility to ciprofloxacin were detected in 31 isolates (73.8%), with 27 of these isolates also exhibiting low MIC values to ciprofloxacin (ECOFF > 0.5 mg/L) (Table 2). A total of four discrepancies (three major and one very major error) were detected between the phenotypic and genotypic profiles in 4 out of 42 (9.5%) of the isolates tested (Table 3). The most common mutation in the *gyr*A gene (T86I) detected was present in 29 of 42 isolates (Table 2).

#### 3.3.3. Resistance to Tetracyclines

Reduced susceptibility to tetracycline determined by the presence of tet variants was detected in six isolates, with five of these determined as isolates with acquired resistance, showing MIC values to tetracycline higher than the expected ECOFF (ECOFF > 1 mg/L) (Table 2). A total of two discrepancies were detected between phenotypic and genotypic profiles; both were classified as very major errors (Table 3).

#### 3.3.4. Resistance to Aminoglycosides

Reduced susceptibility to aminoglycosides (gentamicin and/or streptomycin) is predicted by the presence of two genes: aac(3)—XI and ant(6)—Ia, with ant(6)—Ia being associated with resistance to streptomycin and aac(3)—XI being associated with resistance to an unknown aminoglycoside. No discrepancies were found between the phenotypic and the genotypic profiles for gentamicin resistance in any of the 42 isolates tested in this research. Streptomycin resistance was predicted to occur by detection of ant(6)-Ia 2 isolates. The isolates were tested only for gentamicin, so we could not compare the genotypic and phenotypic results (Table 2).

## 4. Discussion

The genetic bases of AMR in *C. jejuni* to clinically relevant classes of antimicrobials were previously described [13,14,15,16,17,18,19,20,21,22,23,24,25,26,27,28,29,30,36,37,38,39]. These include mutations in the *gyr*A gene conferring resistance to ciprofloxacin, acquisition of the *tet*(O) gene conferring tetracycline resistance, and mutations in two out of three of the 23S rRNA genes conferring resistance to erythromycin.

In this study, we analysed the genomic data of resistance determinants in *C. jejuni* strains obtained through WGS sequencing, collected from several independent laboratories in Croatia. As the study was designed to analyse specifically human strains of *C. jejuni* and included strains isolated from primarily sterile sites, this study is unique for Croatia and outside its borders, as generally there is a limited number of similar studies focusing on human-derived *C. jejuni* strains.

The prediction strength of WGS analysis in determining AMR was evaluated, comparing the genomic data obtained through WGS to the phenotypic AST results obtained by the broth microdilution method. Once compared, a high correlation between methods was shown (97.5%) as excpected.

The discordances in the results include three strains showing genotypic resistance to ciprofloxacin that were phenotypically classified as strains without acquired resistance, which can be explained by several different single *gyr*A modifications known to be associated with fluoroquinolone resistance in Campylobacter species: Thr86Ile, Asp90Asn, Thr86Lys, Thr86Ala, Thr86Val, and Asp90Tyr. While some forms of mutation confer high-level resistance to this group of antimicrobials, others do not play an important role in quinolone resistance, allowing such isolates to present themselves as phenotypically sensitive during in vitro susceptibility testing [37,38]. Moreover, detected resistance determinants do not always confer a resistant phenotype. There was also one strain in our study genotypically susceptible to ciprofloxacin and two to tetracycline, but they tested phenotypically as a strain with acquired resistance to mentioned antibiotics. These findings can be explained by the existence of multiple mechanisms of resistance to ciprofloxacin and tetracycline, which include a decrease in the outer membrane permeability and efflux systems, and not only *gyr*A modifications or the existence of *tet*(O).

Genotypically tested strains in our study showed antimicrobial resistance in only three classes of tested antimicrobial agents: streptomycin and an unknown aminoglycoside, ciprofloxacin (fluoroquinolone), and tetracycline (tetracycline). The most prevalent resistance determinant in this study was the mutation in the *gyr*A gene (T86I) which was detected in 29 of 42 isolates. This is also the most prevalent mutation described worldwide [8].

Resistance of 77.5% to ciprofloxacin (fluoroquinolone) was followed by 14% of tetracycline resistance, and 7% of streptomycin (aminoglycoside) resistance. There were no isolates in this study classified as multidrug-resistant (exhibiting resistance to three or more classes of antimicrobials), which can be observed in literature nowadays, as *C. jejuni* strains with multiple resistance patterns to several classes of antibiotics are emerging and are also described in Croatia [39,40].

There were nine strains found as CIP-TET resistant, and these were strongly associated with the specific region, originating from the region of Pula and Split. This could not be explained through clonal spreading as strains were of diverse STs.

Resistance data presented in this study do not represent resistance rates for *C. jejuni* in Croatia but are very much in accordance with national data. Croatian national resistance rates in 2020 were 71% for ciprofloxacin and 1% for erythromycin [41], and this was reflected in our collection of isolates. The high concordance between phenotypic and genotypic resistance patterns demonstrated in this study indicates that data obtained through national AMR surveillance programs are reliable and represent a good dataset for clinical and epidemiological use. All stated also pose WGS as a valuable and reliable tool in predicting antimicrobial resistance. Sequentially the implementation of WGS as a routine tool in the surveillance of antibiotic resistance could provide information on the early emergence and spread of AMR and further inform timely policy development on AMR control.

In general, human *C.jejuni* infection is a self-limiting disease and antimicrobial therapy is not routinely recommended. However, in case of severe and prolonged symptoms, the treatment of choice includes ciprofloxacin or a macrolide. High rates of ciprofloxacin resistance also observed in this study are therefore of special concern. Lower resistance to invasive strains observed in this study may be due to the more clonal origin of invasive isolates and the predominance of the ST-49 linked to the wilde-type phenotype.

*C. jejuni* is recognised to be a highly diverse pathogen, represented currently by around 11,884 distinct STs and over 45 clonal complexes [42]. The collection of strains presented in this study shows a polyclonal genetic background comprised of 13 clonal complexes among which ST-51, ST-49, and ST-22 predominate, which is in line with ST-51 being among the top ten STs isolated in Europe [42,43].

The high diversity in genotypical presentation described in our study is in accordance with the other studies on MLST sequence types of *C. jejuni* isolated specifically from humans. Studies performed in Europe showed that predominant STs circulating in patients with campylobacteriosis, i.e., ST21, ST22, ST45, ST48, ST53, ST257, and ST267 [44,45,46,47,48,49,50], out of which only ST22 strains were identified in our study. Referring to the data outside Europe, several other *C. jejuni* MLST variants were detected, which were not identified in Croatia during this study [42,43,48,49,51,52]. The frequency of particular genotypes varies between countries and is influenced by multiple factors. Possible factors influencing this genotypic diversity in the epidemiological presentation include variations in food sources, animal reservoirs, seasons, and different levels of zoonotic transmissions and rates of horizontal gene transfer [53,54].

In our study, ciprofloxacin resistance was strongly associated with ST-51, ST-49, and ST-22 strains. Diverse findings were described throughout the studies. The Fiedoruk group detected genetic determinants associated with fluoroquinolones resistance in just 32% of the ST-51 strains [55].

*C. jejuni* strains from primary sterile specimens are shown to be genetically more uniform, showing an epidemiologically more homogenous pattern, as 50% of them belong to ST-49. As can be seen through the minimum spanning tree and dendrogram data, there are no epidemiologically relevant differences between strains isolated from different parts of Croatia.

WGS proved to be a good tool for comprehensive AMR characterization highly concordant with phenotypic AST.

## Figures and Tables

**Table 1:** Identified MLST STs and CCs of tested strains.

SAMPLE Nr.	MLST_ST	MLST_CC	aspA	glnA	gltA	glyA	pgm	tkt	uncA
OS01	2364	ST-353 complex	14	249	5	2	11	3	6
PU01	51	ST-443 complex	7	17	2	15	23	3	12
PU03	9897	/	2	21	12	62	11	67	6
PU04	3335	ST-206 complex	62	4	5	10	2	1	5
PU05	42	ST-42 complex	1	2	3	4	5	9	3
PU06	2086	ST-206 complex	2	4	5	25	11	1	5
PU07	354	ST-354 complex	8	10	2	2	11	12	6
PU09	464	ST-464 complex	24	2	2	2	10	3	1
PU10	6532	ST-42 complex	346	2	3	4	5	9	3
PU11	305	ST-574 complex	9	53	2	10	11	3	3
ST10	22	ST-22 complex	1	3	6	4	3	3	3
ST11	6175	ST-21 complex	2	1	5	10	608	1	5
ST12	824	ST-257 complex	9	2	2	2	11	5	6
ST2	4878	ST-464 complex	7	2	2	2	10	3	1
ST3	2036	ST-353 complex	7	17	52	10	11	3	6
ST4	51	ST-443 complex	7	17	2	15	23	3	12
ST6	4878	ST-464 complex	7	2	2	2	10	3	1
ST7	7355	ST-353 complex	8	17	5	2	10	59	23
ST8	51	ST-443 complex	7	17	2	15	23	3	12
ST9	51	ST-443 complex	7	17	2	15	23	3	12
ZG01	19	ST-21 complex	2	1	5	3	2	1	5
ZG02	19	ST-21 complex	2	1	5	3	2	1	5
ZG03	50	ST-21 complex	2	1	12	3	2	1	5
ZG04	19	ST-21 complex	2	1	5	3	2	1	5
ZG06	822	ST-21 complex	2	1	79	3	2	1	5
ZG07	2787	ST-21 complex	2	1	5	3	340	1	5
ZG08	22	ST-22 complex	1	3	6	4	3	3	3
ZG10	52	ST-52 complex	9	25	2	10	22	3	6
ZG11	51	ST-443 complex	7	17	2	15	23	3	12
ZG12	50	ST-21 complex	2	1	12	3	2	1	5
ZG13	51	ST-443 complex	7	17	2	15	23	3	12
ZG14	51	ST-443 complex	7	17	2	15	23	3	12
ZGI01	22	ST-22 complex	1	3	6	4	3	3	3
ZGI02	49	ST-49 complex	3	1	5	17	11	11	6
ZGI03	51	ST-443 complex	7	17	2	15	23	3	12
ZGI04	49	ST-49 complex	3	1	5	17	11	11	6
ZGI05	49	ST-49 complex	3	1	5	17	11	11	6
ZGI06	22	ST-22 complex	1	3	6	4	3	3	3
ZGI07	Unknown	Unknown	2	1	79	3	23	1	5
ZGI08	49	ST-49 complex	3	1	5	17	11	11	6
ZGI09	49	ST-49 complex	3	1	5	17	11	11	6
ZGI10	Unknown	Unknown	/	/	/	15	/	/	12

**Table 2:** Phenotypic and genotypic results of tested strains (RD—resistance determinants, W—wild-type, NW—non-wild-type).

Isolate Nr.	Antimicrobial Agent (Class)											
	Chloramphenicol (Amphenicol)		Erythromycin (Macrolide)		Gentamicin (Aminoglycoside)		Ciprofloxacin (Fluoroquinolone)		Tetracycline (Tetracycline)		Ertapenem (Beta-Lactam)	
	16 mg/L	RD	8 mg/L	RD	2 mg/L	RD	0.5 mg/L	RD	1 mg/L	RD	1 mg/L	RD
OS1	W	-	W	-	W	-	NW		W		W	-
PU01	W	-	W	-	W	-	NW	*gyr*A T86I aca-ata	W		W	-
PU03	W	-	W	-	W	-	NW	*gyr*A T86I aca-ata	NW	*tet* (O/32/O)	W	-
PU04	W	-	W	-	W	-	NW	*gyr*A T86I aca-ata	NW	*tet* (O/32/O)	W	-
PU05	W	-	W	-	W	-	W		W		W	-
PU06	W	-	W	-	W	ant (6)-Ia	NW	*gyr*A T86I aca-ata	NW		W	-
PU07	W	-	W	-	W	-	NW	*gyr*A T86I aca-ata	W		W	-
PU09	W	-	W	-	W	-	NW	*gyr*A T86I aca-ata	NW	*tet* (O/32/O)	W	-
PU10	W	-	W	-	W	ant (6)-Ia	NW	*gyr*A T86I aca-ata	NW	*tet* (O/32/O) with mutation	W	-
PU11	W	-	W	-	W	-	NW	*gyr*A T86I aca-ata	W		W	-
ST10	W	-	W	-	W	-	NW	*gyr*A	NW		W	-
ST11	W	-	W	-	W	-	W	*gyr*A T86I aca-ata	W		W	-
ST12	W	-	W	-	W	-	W		W		W	-
ST2	W	-	W	-	W	-	W		W		W	-
ST3	W	-	W	-	W	-	NW	*gyr*A T86I aca-ata	W		W	-
ST4	W	-	W	-	W	-	NW	*gyr*A T86I aca-ata	W		W	-
ST6	W	-	W	-	W	-	NW	*gyr*A T86I aca-ata	W		W	-
ST7	W	-	W	-	W	-	NW	*gyr*A T86I aca-ata	NW	*tet* (O/32/O)	W	-
ST8	W	-	W	-	W	-	NW	*gyr*A T86I aca-ata	W		W	-
ST9	W	-	W	-	W	-	NW	*gyr*A T86I aca-ata	NW	*tet*(L)	W	-
ZG01	W	-	W	-	W	-	NW	*gyr*A T86I aca-ata	W		W	-
ZG02	W	-	W	-	W	-	NW	*gyr*A T86I aca-ata	W		W	-
ZG03	W	-	W	-	W	-	NW	*gyr*A T86I aca-ata	W		W	-
ZG04	W	-	W	-	W	-	NW	*gyr*A T86I aca-ata	W		W	-
ZG06	W	-	W	-	W	-	W		W		W	-
ZG07	W	-	W	-	W	-	NW	*gyr*A T86I aca-ata	W		W	-
ZG08	W	-	W	-	W	aac (3)-XI	W	*gyr*A	W		W	-
ZG10	W	-	W	-	W	-	W		W		W	-
ZG11	W	-	W	-	W	-	NW	*gyr*A T86I aca-ata	W		W	-
ZG12	W	-	W	-	W	-	NW	*gyr*A T86I aca-ata	W		W	-
ZG13	W	-	W	-	W	-	NW	*gyr*A T86I aca-ata	W		W	-
ZG14	W	-	W	-	W	-	NW	*gyr*A T86I aca-ata	W		W	-
ZGI01	W	-	W	-	W	-	NW	*gyr*A T86I aca-ata	W		W	-
ZGI02	W	-	W	-	W	-	W	*gyr*A T86I aca-ata	W		W	-
ZGI03	W	-	W	-	W	-	NW	*gyr*A T86I aca-ata	W		W	-
ZGI04	W	-	W	-	W	-	W		W		W	-
ZGI05	W	-	W	-	W	-	W		W		W	-
ZGI06	W	-	W	-	W	-	NW	*gyr*A T86I aca-ata	W		W	-
ZGI07	W	-	W	-	W	-	W		W		W	-
ZGI08	W	-	W	-	W	-	W		W		W	-
ZGI09	W	-	W	-	W	-	W		W		W	-
ZGI10	W	-	W	-	W	-	NW	*gyr*A T86I aca-ata	W		W	-

**Table 3:** Comparison between phenotypic and genotypic antimicrobial resistance.

Antimicrobial Agent (Class)	Phenotype Susceptible		Phenotype Resistant	
	Genotype Resistant	Genotype Susceptible	Genotype Resistant	Genotype Susceptible
Chloramphenicol (amphenicol)	0	42	0	0
Erythromycin (macrolide)	0	42	0	0
Gentamicin (aminoglycoside)	0	42	0	0
Ciprofloxacin (fluoroquinolone)	3	10	28	1
Tetracycline (tetracycline)	0	34	6	2
Ertapenem (beta-lactam)	0	42	0	0

## Data Availability

The assembled genomes have been deposited in NCBI under submission number SUB11731231 while awaiting publication.

## Supplementary Materials

The following supporting information can be downloaded at: https://www.mdpi.com/article/10.3390/microorganisms10071410/s1, Table S1: MIC values of tested strains; Table S2: genes connected to a potential AMR identified using ReFinder.
